# A longitudinal genome-wide association study of anti-tumor necrosis factor response among Japanese patients with rheumatoid arthritis

**DOI:** 10.1186/s13075-016-0920-6

**Published:** 2016-01-18

**Authors:** Kyoko Honne, Ingileif Hallgrímsdóttir, Chunsen Wu, Ronnie Sebro, Nicholas P. Jewell, Takeo Sakurai, Masahiro Iwamoto, Seiji Minota, Damini Jawaheer

**Affiliations:** Division of Rheumatology and Clinical Immunology, Jichi Medical University, Shimotsuke, Tochigi Japan; 23andme, Mountain View, CA USA; Research Unit of Gynecology and Obstetrics, Institute of Clinical Research, University of Southern Denmark, Odense, Denmark; Department of Obstertics & Gynecology, Odense University Hospital, Odense, Denmark; Department of Radiology, University of Pennsylvania School of Medicine, Philadelphia, PA 19104 USA; School of Public Health, University of California, Berkeley, CA 94720 USA; Inoue Hospital, Takasaki, Gunma Japan; Center for Genetics, Children’s Hospital Oakland Research Institute, Oakland, CA 94609 USA

**Keywords:** Rheumatoid arthritis, Anti-TNF, GWAS, Japanese, Pharmacogenomics, Pharmacogenetics, MAP3K7, BACH2, GFRA1, WDR27

## Abstract

**Background:**

Studies of Caucasian patients with rheumatoid arthritis (RA) to identify genetic biomarkers of anti-tumor necrosis factor (TNF) response have used response at a single time point as the phenotype with which single nucleotide polymorphism (SNP) associations have been tested. The findings have been inconsistent across studies. Among Japanese patients, only a few SNPs have been investigated. We report here the first genome-wide association study (GWAS) to identify genetic biomarkers of anti-TNF response among Japanese RA patients, using response at 2 time-points for a more reliable clinical phenotype over time.

**Methods:**

Disease Activity Scores based on 28 joint counts (DAS28) were assessed at baseline (before initial therapy), and after 3 and 6 months in 487 Japanese RA patients starting anti-TNF therapy for the first time or switching to a new anti-TNF agent. A genome-wide panel of SNPs was genotyped and additional SNPs were imputed. Using change in DAS28 scores from baseline at both 3 (ΔDAS-3) and 6 months (ΔDAS-6) as the response phenotype, a longitudinal genome-wide association analysis was conducted using generalized estimating equations (GEE) models, adjusting for baseline DAS28, treatment duration, type of anti-TNF agent and concomitant methotrexate. Cross-sectional analyses were performed using multivariate linear regression models, with response from a single time point (ΔDAS-3 or ΔDAS-6) as phenotype; all other variables were the same as in the GEE models.

**Results:**

In the GEE models, borderline significant association was observed at 3 chromosomal regions (6q15: rs284515, p = 6.6x10^−7^; 6q27: rs75908454, p = 6.3x10^−7^ and 10q25.3: rs1679568, p = 8.1x10^−7^), extending to numerous SNPs in linkage disequilibrium (LD) across each region. Potential candidate genes in these regions include *MAP3K7, BACH2* (6q15), *GFRA1* (10q25.3), and *WDR27* (6q27). The association at *GFRA1* replicates a previous finding from a Caucasian dataset. In the cross-sectional analyses, ΔDAS-6 was significantly associated with the 6q15 locus (rs284511, p = 2.5x10^−8^). No other significant or borderline significant associations were identified.

**Conclusion:**

Three genomic regions demonstrated significant or borderline significant associations with anti-TNF response in our dataset of Japanese RA patients, including a locus previously associated among Caucasians. Using repeated measures of response as phenotype enhanced the power to detect these associations.

**Electronic supplementary material:**

The online version of this article (doi:10.1186/s13075-016-0920-6) contains supplementary material, which is available to authorized users.

## Background

Anti-tumor necrosis factor (TNF) agents have been particularly effective in controlling disease activity and joint erosions in rheumatoid arthritis (RA) [1–3]. Nonetheless, a considerable proportion of patients (30–40 %) demonstrate only partial response or no response to these therapies. This complex phenotype of anti-TNF responsiveness has a genetic component, as demonstrated by heritability estimates of 0.59 or more for different clinical measures of response [4]. Several studies conducted mainly in Caucasian populations [5–23] have attempted to identify genetic biomarkers that can reliably predict response to anti-TNF agents in patients with RA. However, there have been no reports of genome-wide association studies (GWAS) for biomarkers of anti-TNF response among Japanese or other East Asian RA patients. Only single nucleotide polymorphisms (SNPs) in the *TRAF1* [24] and *CD84* genes [18] have been examined for association in two small samples of 101 and 151 Japanese patients, respectively. It has been demonstrated for several RA risk loci that there are both similarities and ethnic differences in disease associations between Caucasian and Japanese populations [25–29]. Similarly, although efficacy of anti-TNF therapies appears to be similar in these populations [30, 31], there may be similarities and differences in genetic predictors of anti-TNF response.

Previous studies investigating genetic predictors of anti-TNF response focused on a limited number of candidate genes [5–15, 20–22, 32], and few GWAS have been performed [9, 16–19, 23]. The findings from these different studies have been largely inconsistent. Only a couple of loci (*PTPRC* and *PDE3A-SLCO1C1*) have been associated in more than one study [5, 11, 33], but these associations were not consistently replicated in other studies [8, 14, 20]. Findings at other loci have not been independently replicated [12, 22]. All of these studies used a single assessment of anti-TNF response.

In the present study, we have performed a GWAS of anti-TNF response in a population of Japanese RA patients. In contrast to previous studies, we have used repeated measures of response at 3 and 6 months after initiation of anti-TNF therapy in order to have a more reliable assessment of clinical phenotype over time, and have used longitudinal statistical models to accommodate the repeated measures of the outcome.

## Methods

### Patients

A total of 487 RA patients were recruited from the Jichi Medical University and from Inoue Hospital within the Gunma prefecture in Japan, and prospectively followed for 6 months. All included patients: (1) were of Japanese descent; (2) satisfied the 1987 American College of Rheumatology (ACR) criteria for RA [34]; (3) were not in remission (Disease activity score in 28 joints (DAS28) <2.6) at baseline; and (4) were starting treatment with the anti-TNF agents etanercept (ETN), infliximab (INF) or adalimumab (ADA) for the first time or were switching to a new anti-TNF drug due to inefficacy or adverse events.

### Data and sample collection

#### Study baseline

Detailed clinical and demographic data, including age, sex, disease duration, smoking history, and concurrent treatment with methotrexate and prednisolone, were collected on each patient prior to initiation of anti-TNF therapy (study baseline). A serum sample was collected, and used to measure baseline titers of anti-citrullinated protein antibodies (ACPA), rheumatoid factor (RF) using the RA particle agglutination test (RAPA), and C-reactive protein (CRP) levels. Patients were considered to be seropositive for ACPA and/or RF auto-antibodies if ACPA and RAPA titers were ≥4.5 U/ml and ≥40 units, respectively. Whole blood samples (8.5 ml) were collected from each patient and stored at −80 °C for subsequent DNA extraction.

#### Follow up

All patients were evaluated at 3 and 6 months after initiation of anti-TNF therapy. Tender joint counts (TJC28) and swollen joint counts (SJC28) for 28 joints, patient global scores and CRP levels were assessed at each of these time points. The study protocol was approved by the ethics committee of the Jichi Medical University and by the Institutional Review Board of the Children’s Hospital Oakland Research Institute. Signed informed consent was obtained from each study participant.

### Assessment of response to anti-TNF therapy

RA disease activity was assessed at baseline, 3 months and 6 months by the DAS28 calculated on three variables, including 28-joint counts and CRP, i.e., DAS28CRP3 [35, 36], as follows:

$$ \mathrm{D}\mathrm{A}\mathrm{S}28\mathrm{C}\mathrm{R}\mathrm{P}3=\left(0.56\ast \sqrt{TJC28}\right)+\left(0.28\ast \sqrt{SJC28}\right)+\left(0.36\ast ln\left(CRP+1\right)\ast 1.10\right)+1.15 $$

We will refer to the DAS28CRP3 as DAS28. The change in DAS28 scores from baseline at 3 months and 6 months were calculated as ΔDAS-3 and ΔDAS-6, respectively.

### Genotyping and data cleaning

DNA was extracted from frozen whole blood samples using a standard protocol and a Gentra Autopure system (Gentra Systems, Minneapolis, MN, USA). All samples were genotyped for a total of 1,133,484 SNPs on an Illumina BeadLab1000 platform using the HumanOmni1-Quad BeadChip and the Infinium HD assay (Illumina, San Diego, CA, USA). Genotypes were called using a score threshold of 0.15 in the Illumina BeadStudio software. Data cleaning was performed using the PLINK software [37] (http://pngu.mgh.harvard.edu/~purcell/plink/) SNPs with genotyping rates ≤98 %, minor allele frequencies (MAF) ≤1 %, or not in Hardy Weinberg equilibrium (HWE) (*p* ≤0.0001) were excluded from further analyses due to possible genotyping error. To test for population stratification, principal component analysis (PCA) was performed using the EIGENSTRAT software [38, 39].

### Imputation of genotypes

The cleaned genotypes were phased using the ShapeIT (v2) software [40] and imputation of genotypes was performed using the Impute2 software [41]. All available multi-population haplotypes from the 1000 Genomes haplotypes Phase I integrated variant set (June 2014 release) were used as reference panels both for phasing and imputation, as recommended [42]. The probability distribution of three possible genotypes generated by Impute2 at each imputed SNP was converted to genotypes using the GTOOL software (v 0.7.5) (http://www.well.ox.ac.uk/~cfreeman/software/gwas/gtool.html) and a stringent probability threshold of 0.9 was applied. Imputed SNPs with genotyping rates ≤98 % or MAF <5 % were excluded from subsequent analyses.

### Statistical analyses

#### Variables influencing response to anti-TNF therapy

To identify potential confounder variables that influence patient response to anti-TNF therapy over time, a longitudinal analysis was performed using GEE models to accommodate response at two time-points for each patient and to adjust for within-patient correlation [43]. Repeated measures of the change in DAS28 at 3 and 6 months (i.e., ΔDAS-3 and ΔDAS-6) were used as the outcome variable in the models, and the explanatory variables included baseline DAS28, duration of anti-TNF therapy, age at baseline, RA duration, sex, concurrent methotrexate use (yes/no), concurrent prednisolone use (yes/no), type of anti-TNF agent (ETN, INF or ADA), RAPA (yes/no), smoking status (never/ever) and ACPA seropositivity (yes/no). Each variable was tested for association with repeated measures of the change in DAS28 in univariate and multivariate models.

#### Longitudinal genome-wide association analyses

Longitudinal GEE models were used to investigate associations between each SNP on a genome-wide scale and patient response to anti-TNF therapy over time (response at two time points for each patient). For each SNP, repeated measures of the change in DAS28 at 3 and 6 months (i.e., ΔDAS-3 and ΔDAS-6) were used as the outcome variable in the model, with the SNP being the explanatory variable. Covariates included in the model were baseline DAS28, concurrent methotrexate use (yes/no), type of anti-TNF agent (ETN, INF or ADA) and duration of anti-TNF therapy. These covariates were selected on the basis of their association with change in DAS28 from the GEE models described earlier. Principal components were not adjusted for in the main model because the genomic control inflation factor (λ_GC_) was estimated at 1.001. These analyses were repeated after excluding patients who had mild disease activity at baseline.

All analyses were performed using the STATA package (version 13). *P* values below a non-stringent threshold of 1×10^−6^ were taken as evidence of borderline significant association.

#### Cross-sectional genome-wide association analyses at each time point

Cross-sectional genome-wide association (GWA) analyses were performed using response data from a single time point, at 3 months or at 6 months, as the phenotype in two separate multivariate linear regression models. These models (model 1 and model 2) differed from the GEE models only in terms of the response phenotype: ΔDAS-3 was the outcome variable in model 1, and ΔDAS-6 in model 2. All other explanatory variables and covariates were the same as those in the GEE models. The GWA analyses for models 1 and 2 were performed using the PLINK software.

#### Identifying regions of association

Independent chromosomal regions of association were identified using the LD clumping option within PLINK, based on *p* values and patterns of LD in the data.

## Results

### Patient characteristics

A total of 444 patients had genotype data and data on ΔDAS-3, ΔDAS-6, and covariates available for analysis. Among these, ΔDAS-3 was missing for 3 patients and ΔDAS-6 was missing for 22 patients. The clinical, demographic and treatment characteristics of the patients at baseline are summarized in Table 1. The majority of patients were female (84 %), with RA duration of 8.1±8.5 years, had moderate to severe disease activity at baseline (93 %), were naïve to anti-TNF drugs (94 %), were concurrently being treated with methotrexate (80 %) and were positive for ACPA antibodies (89 %). Overall, response to anti-TNF therapy was significantly better at 6 months than at 3 months (ΔDAS-3 (mean ± SD): 1.50±1.01, ΔDAS-6: 1.72±1.12; *p* = 0.002).

**Table 1:** Patient characteristics at baseline

Characteristic	All patients (n = 444)
Age, years	56.4 ± 12.7
Sex, female	373 (84.0 %)
RA duration (years)	8.1 ± 8.5
Serum CRP (mg/l)	27.6 ± 27.9
Tender joint count	6 (3–11)
Swollen joint count	6 (3–10)
DAS28CRP3 score	4.6 ± 1.0
Disease activity	
Mild (2.6 < DAS28 ≤ 3.2)	31 (7.0 %)
Moderate (3.2 < DAS28 ≤ 5.1)	282 (63.5 %)
Severe (DAS28 > 5.1)	131 (29.5 %)
Anti-TNF therapy	
Etarnercept	172 (38.7 %)
Infliximab	242 (54.5 %)
Adalimumab	30 (6.8 %)
Other medications	
Methotrexate	354 (79.7 %)
Prednisolone	283 (65.1 %)*^a^
Anti-TNF naïve at baseline	419 (94.4 %)
Switching to a new anti-TNF agent at baseline	18 (4.1 %)
Ever smoker	94 (23.4 %)*^b^
ACPA positive	330 (88.7 %)*^c^
RF positive	337 (80.1 %)*^d^

Results are shown as mean ± SD, median (IQR) or number (%). *Data for these variables were not available for all patients. Sample sizes were as follows ^a^435, ^b^402, ^c^372, ^d^421. *RA* rheumatoid arthritis, *CRP* C-reactive protein, Disease Activity Score in 28 joints *DAS28*, *ACPA* anti-citrullinated protein antibodies, *RF* rheumatoid factor

### Variables influencing response to anti-TNF therapy

In univariate and multivariate GEE models, only baseline DAS28 (*p* <0.0005), concurrent methotrexate use (*p* <0.0005) and duration of anti-TNF therapy (*p* <0.0005) were associated with change in DAS28 (Table 2).

**Table 2:** Results from multivariate GEE model to identify predictors of response

Variable	β (95 % CI)	*P* value
Age at baseline, years	−0.003 (−0.01, 0.004)	0.35
RA duration, years	0.001 (−0.01, 0.008)	0.84
Sex, male^a^/female	−0.13 (−0.41, 0.16)	0.39
DAS28 at baseline	0.39 (0.30, 0.49)	<0.0005
Duration of anti-TNF therapy, weeks	0.02 (0.01, 0.03)	<0.0005
RAPA seropositivity, yes/no^a^	−0.07 (−0.39, 0.25)	0.66
ACPA seropositivity, yes/no^a^	0.10 (−0.28, 0.48)	0.61
Smoking status, never^a^/past/current	−0.10 (−0.24, 0.05)	0.18
Concurrent methotrexate use, yes/no^a^	0.43 (0.19, 0.68)	<0.0005
Concurrent prednisolone use, yes/no^a^	−0.08 (−0.28, 0.11)	0.40
Type of anti-TNF therapy, ETN^a^, ADA or INF	−0.08 (−0.18, 0.02)	0.12

*β* regression coefficient. ^a^Reference category for these variables in the generalized estimating equations (GEE) model, *RA* rheumatoid arthritis, *DAS28* Disease Activity Score in 28 joints, *RAPA* rheumatoid arthritis particle agglutination, *ACPA* anti-citrullinated protein antibodies

### Data quality and population stratification

A total of 4,253,138 autosomal SNPs were available for statistical analyses after imputation and after quality control thresholds were applied. Of these, 738,576 had been genotyped, and the rest had been imputed with high accuracy (imputation accuracy score >0.96).

### SNP associations

#### GEE models using repeated measures of change in DAS28 as the phenotype

**Fig. 1:**
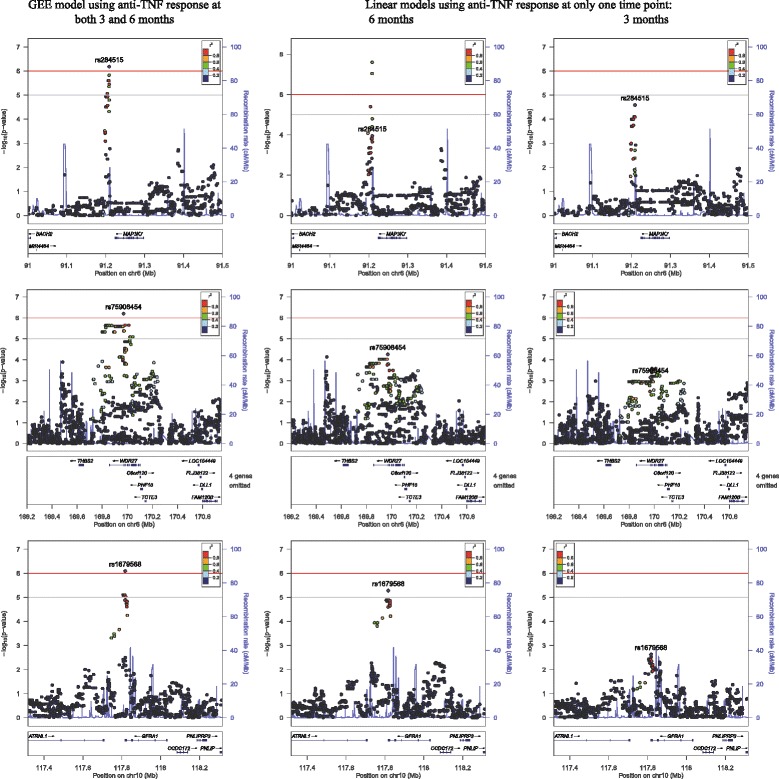
Regions demonstrating significant or borderline significant association with anti-TNF response. The regional plots show three regions with borderline significant association (*p* <1×10^−6^) at 6q15 (*top row*), 6q27 (*middle row*) and 10q25.3 (*bottom row*), based on results from all patients (n = 444) and generalized estimating equations (*GEE*) models using anti-TNF response at two time points, i.e., 3 and 6 months (*left panels*). Association results at the same genomic regions are shown for models using anti-TNF response at a single time point only: 6 months (*middle panels*) and 3 months (*right panels*). Note: at 6 months, another single nucleotide polymorphism (SNP) in linkage disequilibrium with rs284515 the 6q15 locus (i.e., rs284511) was significantly associated with response at 6 months (change in Disease Activity Score at 6 months, ΔDAS-6); this SNP was not associated at 3 months, and was borderline significant in the GEE model. *Chr* chromosome

Three independent genomic regions showed borderline significant (*p* <1×10^−6^) association with repeated measures of change in DAS28 (i.e., both ΔDAS-3 and ΔDAS-6) (Fig. 1); the results for the index SNPs within each region are shown in Table 3. Two of these regions mapped to chromosome 6: one at 6q15 (rs284515: *p* = 6.6×10^−7^) approximately 15 Kb downstream from the *Mitogen-activated protein kinase kinase kinase 7* (*MAP3K7*) gene and 572 Kb upstream from the *Basic leucine zipper transcription factor 2* (*BACH2*) gene, and the other at 6q27 (rs75908454: *p* = 6.3×10^−7^) within a locus containing the *WD repeat-containing protein 27* (*WDR27*) gene. The third locus (rs1679568: *p* = 8.1×10^−7^) mapped to the 3’ untranslated region (UTR) of the *Glial cell line-derived neurotrophic factor family receptor alpha 1* (*GFRA1*) gene at 10q25.3. As shown in Fig. 1, at each locus, numerous SNPs within the region of LD containing the index SNP also showed evidence of association with change in DAS28. Similar results were obtained when the analysis was restricted to patients with moderate or severe disease activity at baseline (n = 413) (see Additional file 1). In addition, the results were again similar when the analysis was restricted only to the subset of patients who were anti-TNF naïve at baseline (n = 419). In both cases though, the significance levels were lower than in the full dataset of 444 patients. Chromosomal regions showing moderate evidence of association (*p* <1×10^−5^) are shown in Additional file 2, and the index SNPs within those regions are listed in Additional file 3.

**Table 3:** Index SNPs from independent chromosomal regions showing borderline significant association with repeated measures of response at 3 and 6 months

SNP	Chr	Position (bp)	Minor allele	MAF	Genotype	N	β (95 % CI)	*P* value	Gene(s) in region of association
rs284515	6	91,208,828	G	0.17	AG/GG	134	0.35 (0.21, 0.49)	6.6×10^−7^	MAP3K7, BACH2
					AG	121	0.33 (0.16, 0.51)	2.1×10^−4^	
					GG	13	0.80 (0.43, 1.18)	2.6×10^−5^	
rs75908454	6	169,970,623	C	0.10	CT/CC	88	0.42 (0.26, 0.59)	6.3×10^−7^	WDR27
					CT	87	0.41 (0.24, 0.59)	2.3×10^−6^	
					CC	1	-	-	
rs1679568	10	117,817,551	A	0.15	AG/AA	124	0.35 (0.21, 0.49)	8.1×10^−7^	GFRA1
					AG	114	0.37 (0.20, 0.54)	1.6×10^−5^	
					AA	10	0.63 (0.24, 1.02)	1.6×10^−3^	

The single nucleotide polymorphisms (*SNP*) with the smallest *p* value (index SNP) in each of the associated regions are shown. β (regression coefficient) represents the increase in change in DAS28 associated with the genotype(s) shown compared to the reference genotype (with no minor alleles). *Chr* chromosome, *MAF* minor allele frequency, *N* number of patients with genotype(s) shown

The *CD84*, *PTPRC*, *PDE3A-SLCO1C1* and *MED15* loci reported to be significantly associated with anti-TNF response in RA in previous GWAS showed no evidence of association in our dataset.

### Cross-sectional analyses at 3 months and at 6 months

When response at only the 3-month or 6-month follow up (ΔDAS-3 or ΔDAS-6) was used as the phenotype instead of ΔDAS at both time points, SNP rs284511 mapping close to and in LD with rs284515 near the *MAP3K7* locus was significantly associated with ΔDAS-6 at the genome-wide significance level (*p* = 2.5×10^−8^). No other SNPs showed significant or borderline significant associations with either ΔDAS-6 or ΔDAS-3.

Of interest, as shown in Fig. 1 and Table 4, compared to the cross-sectional analyses which used response phenotype from a single time point, the GEE models provided increased power to detect associations at loci that demonstrated modest association at both time points, i.e., rs284515, rs75908454 and rs1679568, but not rs284511.

**Table 4:** SNPs showing significant or borderline significant association in the cross-sectional analyses or in the GEE model

					Model 1			Model 2			GEE model		
SNP	Chr	Position (bp)	Minor allele	MAF	Phenotype: ΔDAS-3			Phenotype: ΔDAS-6			Phenotype: ΔDAS-3 and ΔDAS-6		
					N	β	*P*	N	β	*P*	N	β	*P*
rs284511	6	91,208,542	C	0.33	440	0.16	0.01	421	0.41	2.5×10^−8^	443	0.28	1.5×10^−6^
rs284515	6	91,208,828	G	0.17	438	0.35	5.6×10^−5^	419	0.36	1.1×10^−4^	443	0.35	6.6×10^−7^
rs75908454	6	169,970,623	C	0.10	440	0.36	0.0007	421	0.49	5.5×10^−5^	443	0.42	6.3×10^−7^
rs1679568	10	117,817,551	A	0.15	434	0.27	0.002	415	0.44	5.3×10^−6^	437	0.35	8.1×10^−7^

Association results from cross-sectional (models 1 and 2) and longitudinal (generalized estimating equations (GEE) model) analyses - for single nucleotide polymorphisms (SNPs) within regions showing borderline significant association with the GEE model. *MAF* minor allele frequency *ΔDAS-3* and *ΔDAS-6*, change in Disease Activity Scores based on 28 joint counts (from baseline) at 3 months and 6 months, *Chr* chromosome

## Discussion

This is the first GWAS investigating genetic biomarkers of response to anti-TNF therapy in Japanese patients with RA, utilizing a longitudinal approach to examine associations between genome-wide SNPs and repeated measures of anti-TNF response at 3 and 6 months. We found borderline significant association (*p* <1×10^−6^) at three non-correlated regions within our study population, with the associated SNPs mapping to or close to the following genes: *MAP3K7, BACH2* (6q15), *WDR27* (6q27) and *GFRA1* (10q25.3). Each of these regions harbored numerous SNPs demonstrating evidence of association with change in DAS28. Furthermore, the 6q15 locus was significantly associated with response at 6 months (*p* = 2.5×10^−8^), and the association at the *GFRA1* locus represents a replication of a previously reported association among Caucasian patients. We therefore considered these regions worthy of being reported so that they may be investigated further in larger datasets.

The *MAP3K7* gene encodes transforming growth factor beta-activated kinase 1 (TAK1) which is a key regulator in multiple inflammatory signaling pathways [44, 45], including the p38 MAPK and nuclear factor kappa B signaling pathways. TAK1 deficiency leads to reduced pro-inflammatory cytokine production in cultured RA synoviocytes [46]. It is thus an excellent candidate that may influence the effect of anti-TNF agents, as proposed [47], and is already a candidate therapeutic target to block pro-inflammatory pathways in RA [48, 49]. Transcription factor BACH2, on the other hand, appears to be a key negative regulator of effector T cell differentiation, promoting immune homeostasis [50]. In the mouse, it appears to be a super-enhancer repressing a network of genes critical for T cell function [51]. Of interest, variants at the *BACH2* locus have been associated with multiple autoimmune diseases, including RA [52–57]. The GFRA1 protein is a member of the Glial cell line-derived neurotrophic factor (GDNF) receptor family and mediates activation of the RET tyrosine kinase receptor. GDNF is produced by astrocytes in response to pro-inflammatory cytokines including TNFα [58] and appears to suppress interleukin-17 (IL-17)-mediated inflammation via the NF-kappa B pathway [59]. The function of the WDR27 protein has not been established.

The association with the *GFRA1* gene was previously identified by Plant *et al.* [16]. SNP rs7070180 mapping to an intron of the *GFRA1* gene, was associated with anti-TNF response in a cohort of 566 Caucasian RA patients in the UK (*p* = 2.24×10^−4^) and in a meta-analysis including a cohort of 379 additional patients (*p* = 6.42×10^−5^). However, this locus was not reported among the major findings of that study as SNP rs7070180 failed to genotype in one of the cohorts. While this SNP was not genotyped or imputed in our data, several other SNPs within the 3’ untranslated region (UTR) of the *GFRA1* gene were associated with anti-TNF response among our patients. There are no reports of *MAP3K7* being associated with anti-TNF response in RA, although it has been proposed as a good candidate for pathway pharmacogenetics relating to TNF inhibitors [47]. Among the genes of the p38 MAPK network that have been investigated [7, 9, 13], suggestive evidence of an association with *MAP2K6* was reported [9], though not replicated [13]. Other associations for p38 *MAPK* candidate genes were reported in a sample of 1,102 patients using a generously non-stringent significance threshold of *p* <0.1 [7].

A major strength of the present study is the use of repeated measures of anti-TNF response at 3 months and 6 months after treatment was started. Previous studies included clinical response from a single time point in standard linear or logistic regression models [5–9, 11, 12, 16–19]. However, assessment of response at a single point in time may not adequately reflect a patient’s response to therapy, as response may fluctuate over time. Hence, using response data from at least two time points is more reliable and clinically relevant. The longitudinal approach enables the use of repeated measures of response from different time-points for each patient, thus increasing the power to detect an association as we have demonstrated, while taking into account within-patient correlation. Patients with missing data at one time point were still included in the analyses, as the GEE uses all available data. However, while the association with the *MAP3K7* locus achieved genome-wide significance for SNP rs284511 when using anti-TNF response at only 6 months, the lack of association with this SNP at 3 months led to the GEE model only detecting a borderline significant association.

We did not identify any other overlap between our results and previous findings, possibly due to differences in ethnicity, response variable, i.e., two time points vs a single time point, or duration of anti-TNF treatment (3–12 months in previous studies). Further, the lack of consistent findings between previous studies may also have been the result of differences in a number of factors including study design, clinical outcomes examined (DAS28ESR vs DAS28CRP), specific anti-TNF medications used, concomitant disease modifying anti-rheumatic drugs (DMARDs), sex ratios, and small sample sizes in some cases [9, 12, 19]. Another important difference between studies is the heterogeneity in phenotypes introduced by differences in time from baseline to assessment of response ranging from 3 to 12 months in different studies. As seen in our data, treatment duration is significantly associated with response, and should be given due consideration when using multiple datasets for combined analyses to minimize phenotypic heterogeneity or when comparing results between studies. Further, the low significance thresholds used to identify previously reported associations may have led to false positive associations being included. We also cannot exclude the possibility that our findings may include false positives until they can be replicated in independent datasets or that our sample size was not adequately powered to detect some of the previously reported findings.

The present study has a number of limitations. First, the sample size of 444 patients is modest compared to previous GWAS, which combined data from different populations of European ancestry to achieve large sample sizes [16, 18]. Nonetheless, it represents the largest reported sample size for studies of anti-TNF response among Japanese or any East Asian RA population [18, 24]. Given the lack of pharmacogenomics studies examining associations with anti-TNF response among Japanese RA patients, these results represent an important contribution to the field. Although power may be limited due to the modest sample size, this may in part be compensated for by the ethnic homogeneity of the patient population and the use of response data from two time points as described earlier. Second, the response phenotype in our study included response to three different anti-TNF agents, which may have introduced some bias in responder status because a patient who was a non-responder to one drug, might have responded well to another drug. For example, of the 18 patients who had switched to a new anti-TNF drug at baseline due to inefficacy of the previous drug, 11 had a good response to the second line of anti-TNF agent. However, all patients starting a new anti-TNF agent at study baseline - i.e., those who switched and those who were anti-TNF naïve - were followed for 6 months to assess response as is routinely done in clinical practice. Hence, none of them were switched to a new agent for the duration of the study. To mitigate misclassification bias in the response phenotype that might have arisen from inclusion of three different anti-TNF agents in the analysis, we adjusted for the type of anti-TNF agent used. Third, as there is no gold standard measure to evaluate treatment response in RA, we used the DAS28CRP3 as a surrogate to assess disease activity although it may not be a perfect measure of response. The possibility that such a complex phenotype may be associated only with modest genetic effects has been raised [4, 16]. A comparison of the component variables, i.e., tender and swollen joint counts and CRP levels, showed similar trajectories from baseline to 6 months to the composite DAS28 score. This suggests that no major differences in associations would be expected by focusing on components of the DAS28 as the outcome in our dataset. In order to more closely capture variations in patient response over time, we chose to use repeated measures of the change in DAS28 from baseline. We did not categorize the outcome into European League Against Rheumatism (EULAR) responses as this would have led to a reduction in power. Last, the association between a genetic predictor and clinical response could be confounded by factors that influence response. As far as possible, we adjusted for likely confounders associated with change in DAS28 in our dataset. We did not, however, adjust for variations in drug dosage. We had previously reported that response to anti-TNF therapy is influenced by sex in the long term [60], but that these sex differences were not observed during the first 6 months of treatment. Thus, the lack of an association with sex in the present dataset, which was followed for only 6 months, is in agreement with our previous findings.

## Conclusions

In summary, we have identified three chromosomal regions demonstrating significant or borderline significant association with response to anti-TNF therapy among Japanese RA patients. The replication in our Japanese dataset of a previous association with the *GFRA1* locus among Caucasians provides evidence for a trans-ethnic association of this locus with anti-TNF response. We have also demonstrated the importance of including response at more than one time point in order to enhance power to detect associations in such pharmacogenomics studies of RA.

## Additional files

Additional file 1: Figure S1. Regional plots showing association results from GEE models at 6q15, 6q27 and 10q25.3, when the analyses were restricted to patients with moderate or severe disease activity at baseline (n = 413). (PDF 350 kb) Additional file 2: Figure S2. Regions showing *moderate* evidence of association (p < 1x10^−5^) with anti-TNF response (GEE models). (PDF 324 kb) Additional file 3: Table S1. SNPs showing *moderate* evidence of association (p < 1x10^−5^) with anti-TNF response (GEE models). (PDF 183 kb)

## Abbreviations

ΔDAS-3 and ΔDAS-6: change in Disease Activity Scores based on 28 joint counts (from baseline) at 3 months and 6 months
ACPA: anti-citrullinated protein antibodies
ACR: American College of Rheumatology
ADA: adalimumab
BACH2: basic leucine zipper transcription factor 2
bp: base pairs
CRP: C-reactive protein
DAS28: Disease Activity Scores based on 28 joint counts
DMARD: disease modifying anti-rheumatic drug
ETN: etanercept
GDNF: glial cell line-derived neurotrophic factor
GEE: generalized estimating equations
GFRA1: glial cell line-derived neurotrophic factor family receptor alpha 1
GWAS: genome-wide association study
HWE: Hardy Weinberg equilibrium
IL-17: interleukin-17
INF: infliximab
LD: linkage disequilibrium
MAF: minor allele frequency
MAP3K7: mitogen-activated protein kinase kinase kinase 7
PCA: principal component analysis
RA: rheumatoid arthritis
RAPA: rheumatoid arthritis particle agglutination
RF: rheumatoid factor
SJC28: swollen joint counts for 28 joints
SNP: single nucleotide polymorphism
TAK1: transforming growth factor beta-activated kinase 1
TJC28: tender joint counts for 28 joints
TNF: tumor necrosis factor
UTR: untranslated region
WDR27: WD repeat-containing protein 27
