# Supplementary material for: A longitudinal genome-wide association study of anti-tumor necrosis factor response among Japanese patients with rheumatoid arthritis
Source: Arthritis Res Ther. 2016 Jan 18;18:12. doi: 10.1186/s13075-016-0920-6 (PMC4718049; doi:10.1186/s13075-016-0920-6)
Supplement: Additional file 1: Figure S1. — Regional plots showing association results from GEE models at 6q15, 6q27 and 10q25.3, when the analyses were restricted to patients with moderate or severe disease activity at baseline (n = 413). (PDF 350 kb) [file 13075_2016_920_MOESM1_ESM.pdf]

Additional Figure 1

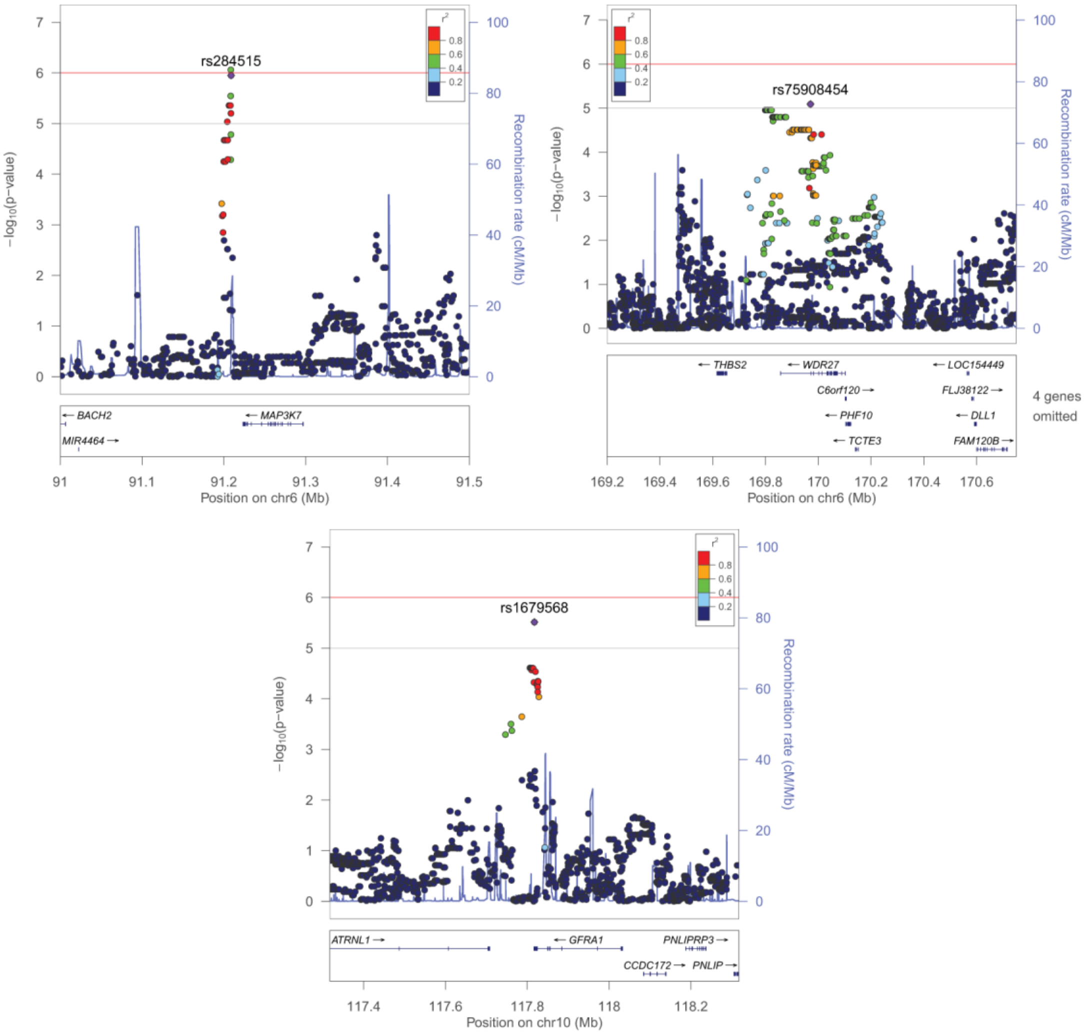

Regional plots showing association results from GEE models at 6q15, 6q27 and 10q25.3, when the analyses were restricted to patients with moderate or severe disease activity at baseline (n=413).
