# Supplementary material for: A longitudinal genome-wide association study of anti-tumor necrosis factor response among Japanese patients with rheumatoid arthritis
Source: Arthritis Res Ther. 2016 Jan 18;18:12. doi: 10.1186/s13075-016-0920-6 (PMC4718049; doi:10.1186/s13075-016-0920-6)
Supplement: Additional file 2: Figure S2. — Regions showing moderate evidence of association (p < 1x10^−5^) with anti-TNF response (GEE models). (PDF 324 kb) [file 13075_2016_920_MOESM2_ESM.pdf]

Additional Figure 2

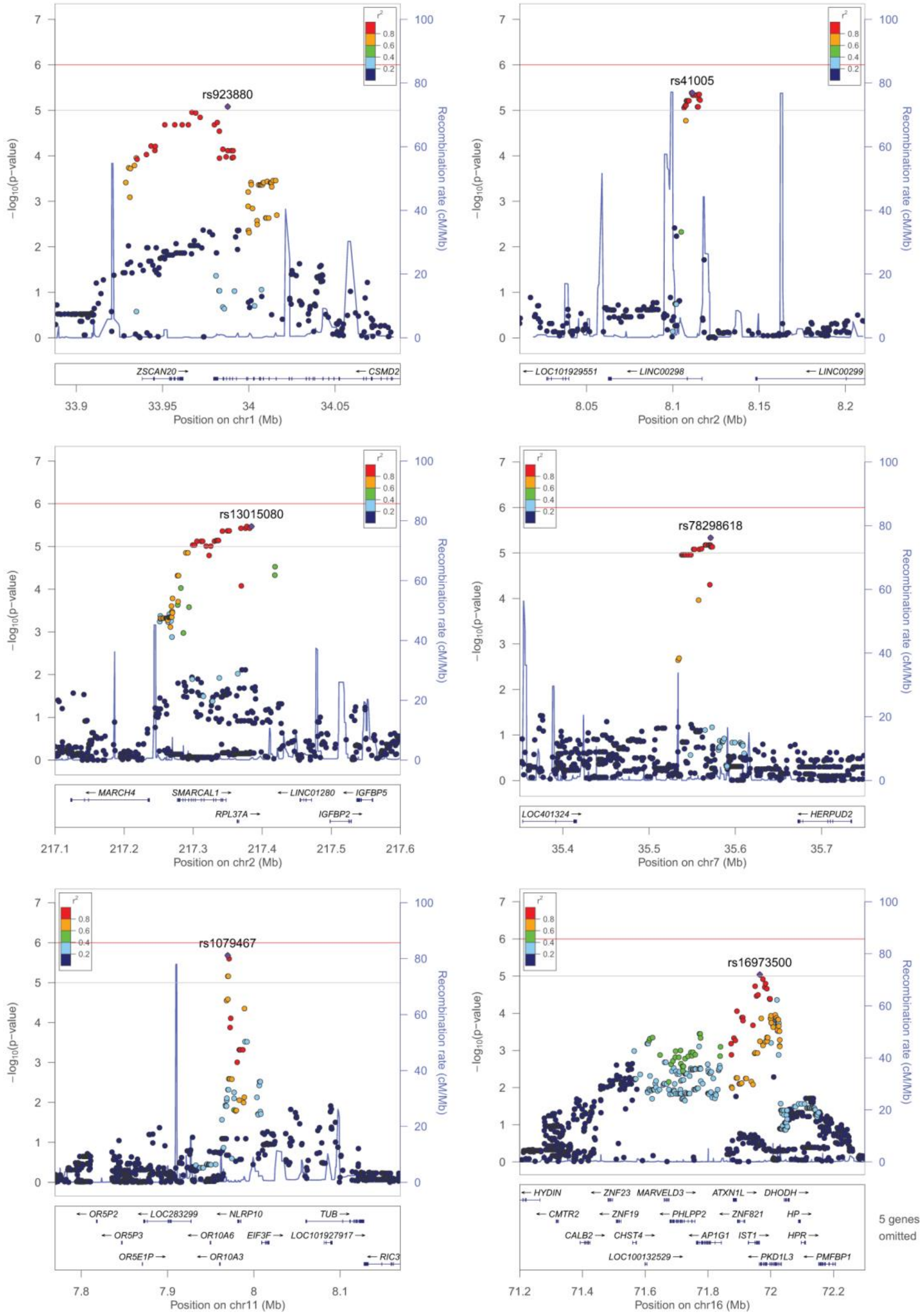

Regions showing moderate evidence of association ($p<1x10^{-5}$) with anti-TNF response (GEE models).
