# Supplementary material for: A longitudinal genome-wide association study of anti-tumor necrosis factor response among Japanese patients with rheumatoid arthritis
Source: Arthritis Res Ther. 2016 Jan 18;18:12. doi: 10.1186/s13075-016-0920-6 (PMC4718049; doi:10.1186/s13075-016-0920-6)
Supplement: Additional file 3: Table S1. — SNPs showing moderate evidence of association (p < 1x10^−5^) with anti-TNF response (GEE models). (PDF 183 kb) [file 13075_2016_920_MOESM3_ESM.pdf]

Additional Table 1 – SNPs showing *moderate* evidence of association ($p<1x10^{-5}$) with anti-TNF response.

					All patients (n=444)		Patients with moderate/severe disease activity at baseline (n=413)		
**SNP**	**Chr**	**Position (bp)**	**Minor allele**	**MAF**	**β (95% CI)**	**P value**	**β (95% CI)**	**P value**	**Gene(s) in region of association**
rs923880	1	33,987,778	C	0.43	-0.25 (-0.36, -0.14)	8.3×10^{-6}			CSMD2
rs41005	2	8,111,231	T	0.33	0.27 (0.16, 0.39)	4.1×10^{-6}			-
rs13015080*	2	217,384,482	A	0.31	*-0.28 (-0.40, -0.16)*	*3.4×10^{-6}*	-0.30 (-0.43, -0.18)	1.1×10^{-6}	SMARCAL1
rs1498103*	5	19,839,503	A	0.46	*0.24 (0.12, 0.35)*	*3.3×10^{-5}*	0.26 (0.15, 0.38)	5.8×10^{-6}	CDH18
rs78298618	7	35,571,257	T	0.07	-0.48 (-0.69, -0.28)	4.6×10^{-6}			HERPUD2
rs1079467	11	7,969,711	C	0.26	0.29 (0.17, 0.41)	2.1×10^{-6}			NLRP10
rs16973500	16	71,965,196	T	0.38	0.24 (0.13, 0.34)	9.1×10^{-6}			PKD1L3, IST1, ATXN1L

Abbreviations: Chr: chromosome; MAF: Minor allele frequency; β: regression coefficient; 95% CI: 95% confidence interval.

* In these 2 regions, increased significance was achieved in the patient subset where those with mild disease activity at baseline had been excluded (n=413).
